# Integrated Bulk Segregant Analysis, Fine Mapping, and Transcriptome Revealed QTLs and Candidate Genes Associated with Drought Adaptation in Wild Watermelon

**DOI:** 10.3390/ijms25010065

**Published:** 2023-12-20

**Authors:** Ahmed Mahmoud, Rui Qi, Xiaolu Chi, Nanqiao Liao, Guy Kateta Malangisha, Abid Ali, Mohamed Moustafa-Farag, Jinghua Yang, Mingfang Zhang, Zhongyuan Hu

**Affiliations:** 1 Institute of Vegetable Science, Zhejiang University, Hangzhou 310058, China; 11716103@zju.edu.cn (A.M.); qirui@zju.edu.cn (R.Q.); 22216178@zju.edu.cn (X.C.); lnqiao@zju.edu.cn (N.L.); malangisha@zju.edu.cn (G.K.M.); 11516099@zju.edu.cn (A.A.); yangjinghua@zju.edu.cn (J.Y.); mfzhang@zju.edu.cn (M.Z.); 2 Hainan Institute of Zhejiang University, Yazhou District, Sanya 572025, China; 3 Key Laboratory of Horticultural Plant Growth, Development & Quality Improvement, Ministry of Agriculture, Hangzhou 310058, China; 4 Horticulture Research Institute, Agricultural Research Center, 9 Cairo University St, Giza 12619, Egypt; m_m_kamel2005@gdaas.cn

**Keywords:** drought tolerance, genetic resources, wild watermelon, quantitative trait loci (QTL) mapping, transcriptome, genomics-assisted breeding

## Abstract

Drought stress has detrimental effects on crop productivity worldwide. A strong root system is crucial for maintaining water and nutrients uptake under drought stress. Wild watermelons possess resilient roots with excellent drought adaptability. However, the genetic factors controlling this trait remain uninvestigated. In this study, we conducted a bulk segregant analysis (BSA) on an F_2_ population consisting of two watermelon genotypes, wild and domesticated, which differ in their lateral root development under drought conditions. We identified two quantitative trait loci (*qNLR_Dr. Chr01* and *qNLR_Dr. Chr02*) associated with the lateral root response to drought. Furthermore, we determined that a small region (0.93 Mb in *qNLR_Dr. Chr01*) is closely linked to drought adaptation through quantitative trait loci (QTL) validation and fine mapping. Transcriptome analysis of the parent roots under drought stress revealed unique effects on numerous genes in the sensitive genotype but not in the tolerant genotype. By integrating BSA, fine mapping, and the transcriptome, we identified six genes, namely L-Ascorbate Oxidase (AO), Cellulose Synthase-Interactive Protein 1 (CSI1), Late Embryogenesis Abundant Protein (LEA), Zinc-Finger Homeodomain Protein 2 (ZHD2), Pericycle Factor Type-A 5 (PFA5), and bZIP transcription factor 53-like (bZIP53-like), that might be involved in the drought adaptation. Our findings provide valuable QTLs and genes for marker-assisted selection in improving water-use efficiency and drought tolerance in watermelon. They also lay the groundwork for the genetic manipulation of drought-adapting genes in watermelon and other *Cucurbitacea* species.

## 1. Introduction

In recent years, climate changes have been predicted to cause severe consequences for the entire agroecosystem, including the growing challenge of agricultural water resources [1]. Recent statistics from the Food and Agriculture Organization (FAO) state that more than 60% of the Earth’s inhabitants might face the risk of water shortage by 2025 [2]. Drought is a severe abiotic stress that affects plant growth and development, directly impacting the yield and quality of economic crops [3,4]. Therefore, plant breeders are seeking unique solutions to overcome water deficiency and achieve sustainable agricultural production [5,6]. Since the root system is the first line of defense for maintaining crop survival and growth under drought conditions, breeding new cultivars with a robust root could be a promising approach [7]. Generally, the root’s ability to uptake water depends on the lateral root architectures, starting with lateral root emergence and ending with root hairs [7,8,9,10,11,12,13]. The number of lateral roots (NLR) represents the initial ability of lateral roots to uptake water and nutrients, as the fewer lateral roots there are, the less water and fewer nutrients are absorbed from the soil. Therefore, the much-branched root system, as an essential index for drought tolerance, was previously reported in several plants [7,14,15]; however, this relationship remains unknown in watermelon [16,17].

To date, new varieties with vigorous root systems and drought tolerance are still limited in many terrestrial crops [17]. Wild crops usually possess desirable natural alleles and often display promising characteristics for drought tolerance [18]. Transferring such desirable features to modern cultivars is critical to producing better performance in a stressful environment. This approach requires detecting tolerant germplasms, identifying desirable natural alleles, and then integrating them into cultivated varieties via marker-assisted breeding or genomic selection approaches [19]. Wild watermelon, which originates in the dry areas of Africa, is an important xerophytic crop grown throughout the world [20]. The ancestral genes found in wild watermelons, such as *Citrullus amarus* and *Citrullus colocynthesis*, offer valuable genetic resources for developing new cultivars with stress-resistance traits, particularly a strong root system [15,21,22]. Although previous studies stated that wild watermelons possess resilient roots with unique drought resistance, the genetic factors controlling this trait remain unclear [23,24,25,26]. Moreover, the difficulties in the root phenotyping and hunting of multiple quantitative trait loci (QTLs)/genes related to desirable root traits remain challenges [22].

Wild watermelon was described as exhibiting several drought-adaptation mechanisms with three main strategies: avoidance, tolerance, and escape [26]. Root and shoot architecture responses contribute to avoidance by maintaining water uptake–loss homeostasis, while tolerance is associated with transcriptomic, proteomic, and metabolic regulation, and the escape strategy is related to the shortening of the plant life cycle [26]. Several drought-mechanism studies and germplasm screenings based on leaves and roots’ responses in watermelon were reported; however, no genetic mapping investigations have been conducted yet [4,23,24,25,27,28,29,30,31,32,33,34,35,36,37,38,39,40,41,42,43,44,45,46]. A strong drought-tolerance ability has mainly been observed in *Citrullus amarus*, *Citrullus colocynthis* [4,27,36,38,39,41], and, in some cases, wild *Citrullus lanatus* [23,28,37,45,46]. Interestingly, enhanced [23,28,38,41] and inhibited [37] root growth were defined as drought-adaptation strategies in watermelon. Both short- and long-term (water withholding or Polyethylene Glycol (PEG) treatments) drought stress in soil or hydroponics under controlled (growth chambers), greenhouse, and field conditions at the seedling stage (1–5 leaves) were examined [4,23,27,28,36,37,38,39,41,43,44,45,46]; however, no investigation has been conducted at the post-germination stage. Specifically, four genes were described as associated with drought-stress adaptation in watermelon, namely drought-induced polypeptide (DRIP-1) [24,34], Ran GTPase CLRan1 [23,28], Cytochrome b561 (CLb561A and CLb561B) [32], and Metallothionein Type-2 (*CL*MT2) [33].

Conventional selection in plant breeding is a laborious process due to several rounds of inbreeding and the large number of offspring per phenotype, especially when the target trait is controlled by polygenes [47]. Alternatively, marker-assisted selection provides an excellent breeding method [48]. This strategy is achieved by identifying markers that enable high-throughput genotyping for specific traits at the seedling stage [48,49]. Genetic variations can be identified via QTL mapping using bi-parental populations or genome-wide association study (GWAS) in natural populations [50]. Root traits are primarily controlled by several genes and often differ due to environmental changes [51,52]. Most QTLs related to root traits contributing to drought tolerance were discovered in monocots, mainly in cereals, such as root length, biomass, root number, seminal root angle and length, deep root growth, root diameter, root branching, and aquaporin activity [7,16,53]. In contrast, fewer efforts were published concerning drought-tolerance-related root traits in dicots, mainly in legumes [17]. Although the NLR-associated QTLs have been discovered in lentil [54], soybean [55], and lettuce [56], among dicots, the QTLs/genes related to this trait in response to drought stress in watermelon have not been well explored yet.

The aim of this work was to elucidate the genetic control of lateral root growth in watermelon exposed to drought stress. In this study, for the first time, we report two QTLs associated with the NLR discrepancy in response to drought using the bulk segregant analysis (BSA) approach in watermelon. Additionally, the transcriptome analysis has enhanced our understanding of the molecular regulation of root growth under drought stress. Furthermore, the integrated QTL mapping, fine mapping, and RNA-sequencing (RNA-seq) have revealed candidate genes within the detected QTLs. Overall, this study provides new insights into the genetic regulation of root growth in response to drought, which can aid in developing high water-use efficiency breeding in *Cucurbitaceae* crops.

## 2. Results

### 2.1. Root Phenotyping of Watermelon Accessions, Parental Line Selection, and Mapping Population Screening under Drought Stress

**Figure 1:**
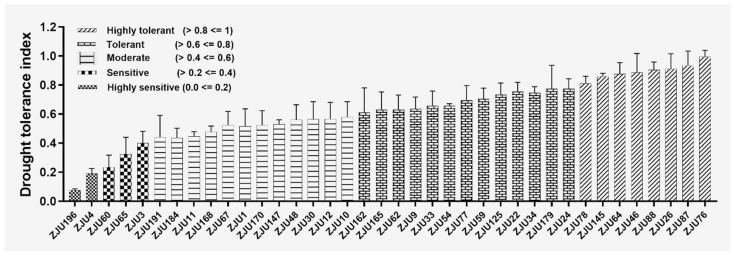
Drought tolerance index (DTI) based on the number of lateral roots (NLR) in 38 watermelon accessions exposed to 15% Polyethylene Glycol (PEG, drought) and distilled water (CK, control) for 4 days in pouches. The DTI was calculated as the ratio between NLR with drought and CK treatments. The tested genotypes were categorized into five groups based on their DTI. The plotted values are averages of three independent biological replications (nine plants/replication/treatment).

The preliminary experiments performed at the seedling stage in pouches indicated that the suitable condition for phenotyping watermelon root response to drought stress was 4 days treatment with 15% PEG6000 (Figure S1). Further, the drought tolerance index (DTI) was calculated based on the NLR values of 38 watermelon accessions. This analysis divided the tested genotypes into five categories, namely highly sensitive (2 accessions), sensitive (3 accessions), moderate (12 accessions), tolerant (13 accessions), and highly tolerant (8 accessions) (Figure 1). From these, the highly sensitive accession (ZJU196) and the tolerant genotype (ZJU076) were selected as excellent parents for QTL mapping.

**Figure 2:**
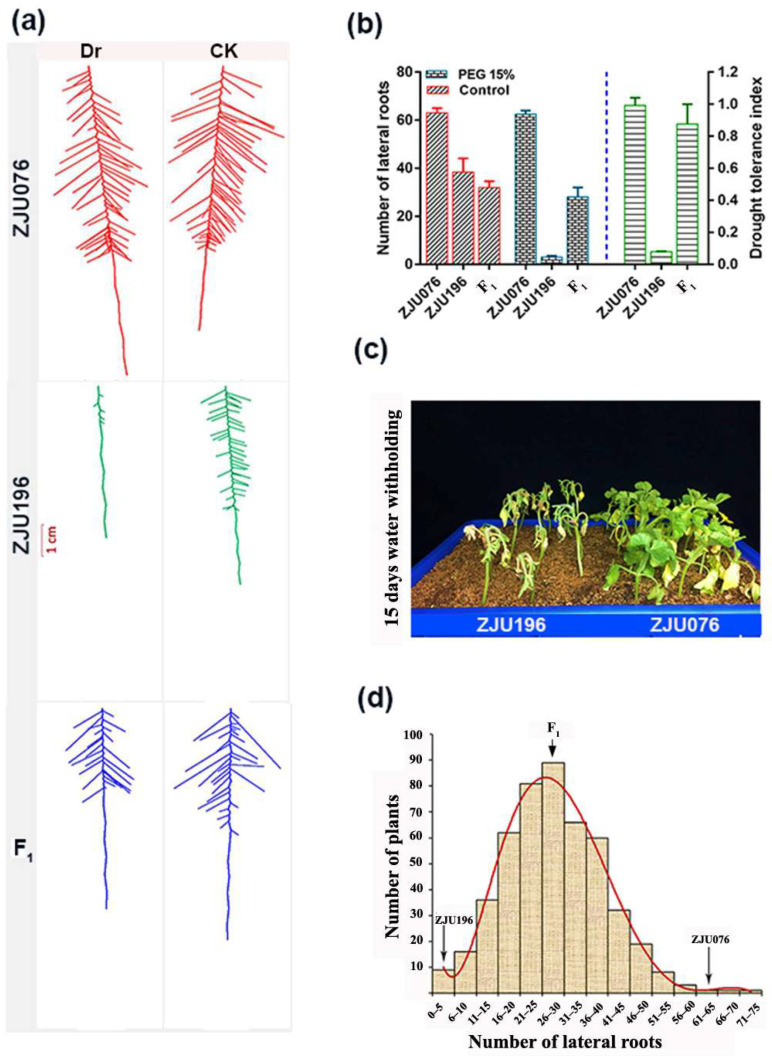
Root phenotyping and validation of drought tolerance in the parents, F_1_ and F_2_ individuals. (**a**) Root phenotypes of ZJU076 (tolerant), ZJU196 (sensitive), and F_1_ after 4 days of growth with 15% PEG6000 (drought, Dr) and distilled water (control, CK). (**b**) Drought tolerance index (DTI) of the parents and F_1_. Values are means ± S.D. (n = 18). (**c**) After 15 days of water withholding (the final soil water content ≈ is 2%), (**d**) Frequency distribution of the number of lateral roots (as drought tolerance indicator) among 484 F_2_ individuals exposed to 15% Polyethylene Glycol (PEG) for four days.

The DTI in ZJU076 (tolerant, 0.99) was higher than that of ZJU196 (sensitive, 0.07) by 14.14-fold (Figure 2a,b & Figure S2). With 15% PEG exposure, the average number of lateral roots was 62.5 in the tolerant genotype (ZJU076) and 2.7 in the sensitive (ZJU196) (Figure 2a,b & Figure S2). Significant differences in the survival rate of the two genotypes in response to water withholding in soil (with a final water content of 2%) for 15 days were confirmed (Figure 2c). Additionally, one week after rehydration, the seedlings phenotype of the tolerant parent exhibited green leaves and viable stems compared to the sensitive parent, supporting a strong tolerance of ZJU076 to drought stress (Figure S3). The F_1_ individuals showed intermediate NLR (28 roots) with 15% PEG in pouches, and their DTI was close to the tolerant parent (0.88; Figure 2a,b). Moreover, the NLR of 484 F_2_ individuals ranged from 0.0 to 75.0 roots and presented a normal distribution (Figure 2d), suggesting that the drought tolerance in this population is likely to be a quantitative trait controlled by polygenes.

### 2.2. Identification and Fine-Mapping of the QTLs Associated with the Drought Adaptation in Watermelon

BSA analysis was conducted to identify the QTLs associated with drought tolerance in watermelon. The Illumina HiSeq4000 sequencing of the ZJU196 (sensitive), ZJU076 (tolerant), high pool, and low pool generated 32.9488 giga-base pairs (Gb) of clean data. The majority of the data presented a high quality, with Q20 ≥ 97.22% and Q30 ≥ 92.94%, and the GC content ranged from 36.39 to 42.75% (Table S1). Approximately 5.472, 5.573, 11.292, and 10.647 Gb clean reads were obtained from ZJU196, ZJU076, the low pool, and the high pool, respectively. Furthermore, the mapping rates of ZJU076, ZJU196, low, and high pools were 68.23, 88.92, 94.97, and 95.33%, respectively.

**Figure 3:**
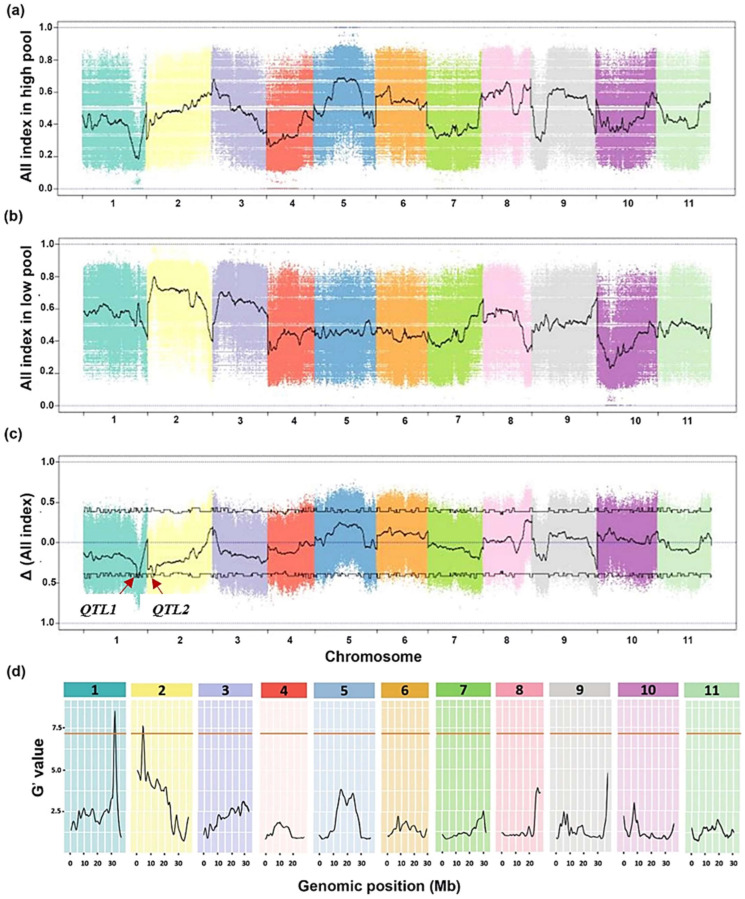
Bulk segregant analysis (BSA) results of the drought tolerance in watermelon. (**a**) The single nucleotide polymorphism (SNP) index of the high pool, (**b**) the SNP index of the low pool, and (**c**) the delta SNP index values used for the association analysis. The x and y axes show the 11 chromosomes of watermelon and the SNP index, respectively. The curved line indicates the fitted SNP index or delta SNP index. The horizontal line indicates the association threshold with FFN of a 95% confidence interval. (**d**) Major QTLs for drought tolerance in watermelon detected by the G prime (G’) method. The *QTL1*, and *QTL2* represent *qNLR_Dr. Chr01* and *qNLR_Dr. Chr02*, respectively.

To detect the QTLs, we calculated and plotted the single nucleotide polymorphism (SNP) indexes of the low and high pools and delta SNP (ΔSNP) to the genome position of watermelon (Figure 3). The QTL-seqr analysis revealed two QTLs associated with NLR-based drought tolerance on chromosome 1 (*Cla97Chr01: 31,601,839–32,694,379*) and chromosome 2 (*Cla97Chr02: 3,943,142–4,743,502*) with an approximate peak ΔSNPs of −0.43 and −0.40, respectively, at a threshold of 95% (Figure 3c; Table S2). The region lengths of the QTL1 and QTL2 were 1.09 and 0.80 mega-base-pairs (Mb), involving 134 and 86 genes, respectively (Table S2). These candidate regions also presented G prime (G’) values above the threshold (Figure 3d; Table S2). Accordingly, we designated the two candidate QTLs associated with drought tolerance as *qNLR_Dr. Chr01* (qtl of the number of lateral roots associated with drought tolerance on Chr01) and *qNLR_Dr. Chr02*.

**Figure 4:**
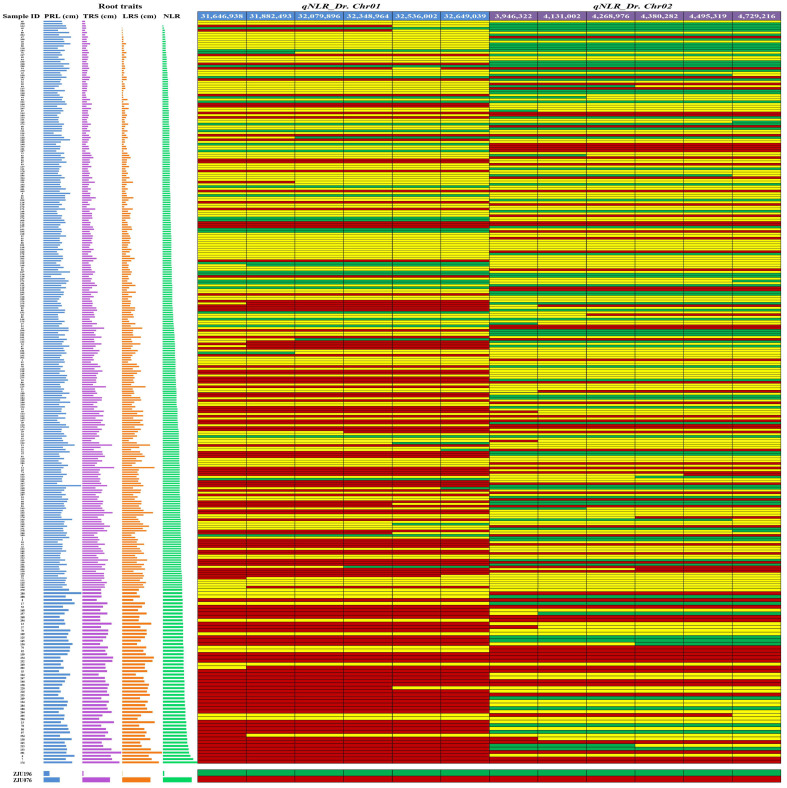
Validation of the detected QTLs through haplotype analysis using six Kompetitive allele specific PCR (KASP) markers for each QTL in 305 F_2_ individuals. Green and red indicate homozygous segments from sensitive and tolerant parents, respectively. Yellow indicates heterozygous segments. The sidebars (on the left) represent the root traits of F_2_ individuals: primary root length (PRL), total root system (TRS), lateral root system (LRS), and the number of lateral roots (NLR). The single nucleotide polymorphism (SNP) data are arranged in ascending order from top to bottom based on the NLR values. Zooming in will make the sample IDs and root trait values much more visible.

To validate the detected QTLs, the SNP haplotype analysis of 305 F_2_ individuals was conducted using twelve Kompetitive allele specific PCR (KASP) markers (six/QTL) designed in the *qNLR_Dr. Chr01* and *qNLR_Dr. Chr02* regions (Figure 4). We arranged the genotyping data from top to bottom ascendingly depending on the NLR. Furthermore, the NLR and the other root traits, including the total root system and the lateral root system, were observed and largely co-distributed throughout the sidebars, while the primary root length did not show any clear association with the genotyping data in this population (Figure 4 & Table S3). In the *qNLR_Dr. Chr01* region, we observed most homozygous segments from the tolerant parent gathered at the lower half of the heatmap, while most homozygous segments from the sensitive parent were located at the upper half (Figure 4 & Table S3). In the *qNL_Dr. Chr02* region, the homozygous segments from the sensitive and tolerant parents were distributed across the heatmap, with many sensitive segments at the top and numerous tolerant segments at the bottom (Figure 4 & Table S3). These results support a high association of *qNLR_Dr. Chr01* with drought tolerance compared to *qNLR_Dr. Chr02*.

**Figure 5:**
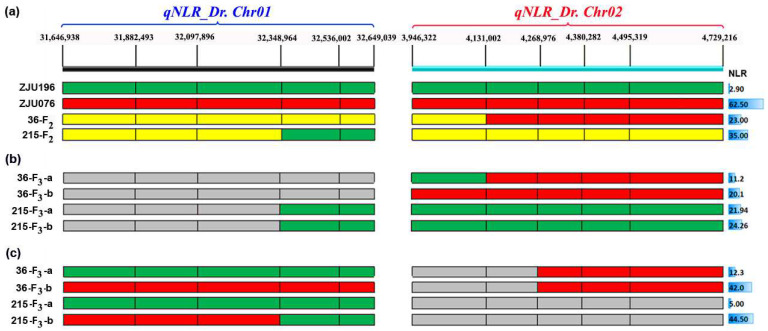
Fine mapping of the detected QTLs with 12 Kompetitive allele specific PCR (KASP) markers. (**a**) Parent genotypes and two F_2_ recombinants in the target regions. The left part represents *qNLR_Dr. Chr01*, while the right part shows *qNLR_Dr. Chr02*. (**b**) The recombinant offspring individuals of F_2_ are sorted based on *qNLR_Dr. Chr02*. (**c**) The recombinant offspring individuals of F_2_ are sorted based on *qNLR_Dr. Chr01*. The F_2_ offspring were classified into two groups based on the segment origins. Red indicates the homozygous ZJU076 (tolerant) segment, green indicates the homozygous ZJU196 (sensitive) segment, yellow shows the heterozygous region, and gray represents mixed (involved individuals similar to the parents or heterozygous). The average number of lateral roots (NLR) of each family was calculated from ten individuals.

To fine-map the target intervals, two F_2_ recombinants around the detected QTLs were screened from 100 F_2_ individuals using 12 KASP markers (Figure 5a). F_2:3_ individuals were divided into two groups (a and b) for each recombinant based on the allelic similarity with the parents (Figure 5a). When the F_2:3_ individuals were classified into two groups based on their genotypes of *qNLR_Dr. Chr02*, regardless of *qNLR_Dr. Chr01* genotypes, we did not observe a clear difference in NLR between the F_2:3_ groups of the used recombinants compared to the parents (Figure 5b). However, when we classified the F_2:3_ individuals into two groups based only on *qNLR_Dr. Chr01* genotypes, the F_3_-a group of both 36 and 215 recombinants showed similar NLR to the drought-sensitive parent (ZJU196), while the F_3_-b group of both recombinants exhibited the NLR close to the drought-tolerant parent (ZJU076; Figure 5c). This result suggests that the *qNLR_Dr. Chr01* might play a stronger role in drought tolerance compared to *qNLR_Dr. Chr02*. Furthermore, the recombination in the 215-F_2_ offspring of *qNLR_Dr. Chr01* helped us to narrow down the candidate *qNLR_Dr. Chr01* to the left region of SNP *Chr01_32348964*, with a chromosome length of 0.93 Mbp (*Chr01: 31601839-32348964*).

### 2.3. Transcriptome Analysis of the Watermelon Root under Drought Stress

To determine potential genes implicated in drought response, we conducted RNA sequencing for ZJU076 (tolerant) and ZJU196 (sensitive) exposed to drought (Dr, 15% PEG6000) and control (CK, 0% PEG6000) treatments for two days (Figure S4). In total, 26.08, 25.87, 26.5, and 25.51 giga-base-pairs (Gb) of clean data were obtained from CK_76 (control_ZJU076), DR_76 (drought_ZJU076), CK_196 (control_ZJU196), and DR_196 (drought_ZJU196), respectively. The majority of the obtained data presented a high quality, with Q20 ≥ 95.08% and Q30 ≥ 87.15%, and the GC content was between 44.72 and 45.23% (Table S4). The alignment results showed that 77.31–79.11% of the sixteen samples’ clean reads were mapped to the watermelon reference genome (Table S4). On average, approximately 37.6 (90.98%) and 39.43 (95.13%) million reads were uniquely mapped to the watermelon reference genome for ZJU076 (tolerant) and ZJU196 (sensitive), respectively (Table S5). The heatmap clustering of the Pearson correlation coefficient between the sixteen samples indicated a robust correlation between replicates of the same treatment (Figure S5), supporting the reliability of the experiment and sample selection. The obtained data presented a high quality and provided a solid basis for detecting associated genes with drought tolerance in watermelon. We compared the two watermelon genotypes through four groups with the drought and control treatments, namely CK_76 vs. Dr_76, CK_196 vs. Dr_196, CK_76 vs. CK_196, and Dr_76 vs. Dr_196. Only the differentially expressed genes (DEGs) with false discovery rate (FDR) < 0.05, |log_2_(Foldchange)| > 1 were considered. The global hierarchical clustering of the DEGs revealed that the drought and control samples were clustered as one group for each variety (Figure S6), implying that the expression patterns of most DEGs in both genotypes were consistent in the control and drought treatments. In total, 11,709 unique DEGs were distinguished in all four comparison groups (Figure S7). We observed 2269 DEGs in CK_76 vs. Dr_76, 5647 in CK_196 vs. Dr_196, 6081 in CK_76 vs. CK_196, and 7852 in Dr_76 vs. Dr_196 (Figure S7). These overlapping significant DEGs could be analyzed through 15 disjointed subgroups; among them, 227 (1.9%), 1200 (10.2%), 979 (8.4%), and 1615 (13.79%) were DEGs specifically found in CK_76 vs. Dr_76, CK_196 vs. Dr_196, CK_76 vs. CK, and Dr_76 vs. Dr_196, respectively (Figure S7). These findings suggest that the drought stress uniquely affected the transcripts of multiple genes in the sensitive genotype compared to the tolerant genotype, supporting a significant difference in the drought tolerance between the selected parents.

Furthermore, we constructed volcano plots for the DEGs to identify the significant changes within the four groups by considering −log_10_ (*p*adj) > 1.3 (*p*adj < 0.05). The data indicated significant changes in the transcript levels within the treatments and genotypes (Figure S8). Due to drought treatment in ZJ076, 1091 and 1178 DEGs were up- and down-regulated, respectively (Figure S8a). In the drought-sensitive (ZJU196), many DEGs were up- and down-regulated (2824 and 2823) in response to drought (Figure S8b). These results implied that the impact of drought stress might be more significant in the drought-sensitive than in the tolerant genotype.

Due to drought stress, a total of 1608 (70.87%) and 4010 (71.01%) DEGs were assigned to Gene ontology (GO) analysis in CK_76 vs. Dr_76 and CK_196 vs. Dr_196, respectively (Figure S9 and Table S6). The catalytic activity (corrected*_p*Value of 1.45 × 10^−7^, includes 913 DEGs) and the oxidoreductase activity (corrected*_p*Value of 4.74 × 10^−5^ includes 543 DEGs) were the most significant molecular functions associated with the drought response in tolerant-ZJU076 (CK vs. Dr) and sensitive-ZJU196 (CK vs. Dr), respectively (Figure S10a,b and Table S6). Moreover, the GO terms related to the cellular components revealed the significance of the cell wall (corrected*_p*Value of 0.003, includes 24 DEGs) and apoplast (corrected*_p*Value of 0.0001, includes 20 DEGs) in response to drought stress in tolerant-ZJU076 (CK vs. Dr) and sensitive-ZJU196 (CK vs. Dr), respectively (Figure S9a,b and Table S6). GO analysis suggests the implication of the oxidation-reduction pathway in the response of the watermelon root to drought stress.

The Kyoto Encyclopedia of Genes and Genomes (KEGG) pathways were analyzed to determine the enriched and significant metabolic pathways relevant to drought stress tolerance in watermelon roots. Among significant pathways with *qvalue* ≤ 0.05, Wnt signaling, starch and sucrose metabolism, retinol metabolism, fatty acid degradation, and cytochrome P450 could be associated with the drought tolerance mechanism in ZJ076 (Figure S10a, Table S7). By contrast, no significant enriched pathways existed in the drought-sensitive genotype (ZJU196) (Figure S10b and Table S7). This finding indicates that significantly induced pathways in ZJU076 (tolerant) might contribute to drought tolerance.

**Figure 6:**
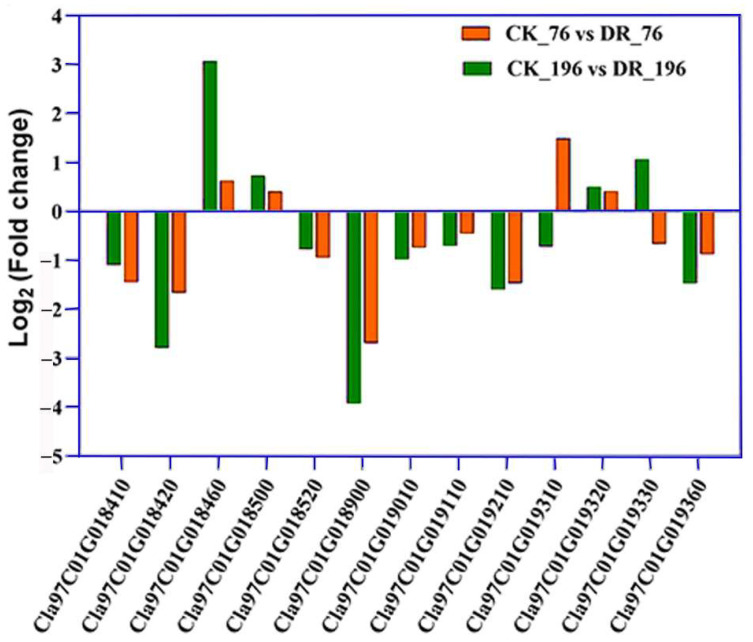
The significant differentially expressed genes (DEGs) within the delimited region of *qNLR_Dr. Chr01*. Log_2_ (fold change) of the 13 DEGs (*p* < 0.05) in CK_76 vs. DR_76 and CK_ 196 vs. D.R._ 196 within the delimited region of *qNLR_Dr. Chr01*.

We integrated the QTL-seq and RNA-seq results to discover the candidate genes. Among the predicted genes in *qNLR_Dr. Chr01* and *qNLR_Dr. Chr02*, only 17 and 10 genes were differentially expressed (*p* < 0.05), respectively, under drought stress (Table S8). In the 0.93 Mb region (*Chr01: 31,601,839-32,348,964*) within the *qNLR_Dr. Chr01* identified by fine-mapping, 13 significant (*P*adj < 0.05) differentially expressed genes were observed (Figure 6). Among them, 12 genes contain at least one nonsynonymous SNP (Table S9). The annotation and functions of these genes have suggested six candidate genes that could be further studied to uncover the underlying mechanism of drought tolerance in watermelon based on the root growth, namely *Cla97C01G018460* (L-Ascorbate Oxidase, AO), *Cla97C01G018500* (Cellulose Synthase-Interactive Protein 1, CSI1), *Cla97C01G018900* (Late Embryogenesis Abundant Protein, LEA), *Cla97C01G019010 (*Zinc-Finger Homeodomain Protein 2, ZHD2), *Cla97C01G019320* (Pericycle Factor Type-A 5, *PFA5*), and *Cla97C01G019330* (bZIP transcription factor 53-like, bZIP53-like) (Table S9).

## 3. Discussion

Water scarcity during the seedling stage undoubtedly affects the functions of different plant parts, especially the root, which is the first plant organ to interact with the environment [17,37,39]. Thus, breeding cultivars with a robust root has become an urgent need in modern agriculture [7]. In the present study, we investigated the drought tolerance index (DTI) of 38 watermelon accessions based on the number of lateral roots (NLR) observed in watermelon seedlings exposed to 15% PEG6000 and control treatments in pouches (Figure 1). Further, we selected an extreme drought-tolerant genotype (ZJU076) and a sensitive genotype (ZJU196) for gene mining related to drought adaptation in watermelon. The wild-tolerant- watermelon (ZJU076), unlike the domesticated-sensitive-genotype (ZJU196), tends to maintain better root growth under normal and water scarcity conditions (Figure 2). Root growth preservation during drought is essential for maintaining the water and nutrient supply to establish the aboveground parts [57]. The consistency of the phenotyping of the two genotypes under water withholding stress in soil (Figure 2c) supported the correlation between long-term drought tolerance and early-stage root response to 15% PEG in pouches (Figure 2a,b). Malambane et al. [27] revealed that watermelon exhibits a similar response pattern in artificial and natural environments under drought stress. The domesticated *C. lanatus*, such as ZJU196 (sensitive), and wild types, such as ZJU076 (tolerant), were recognized in previous studies as drought-sensitive and tolerant species, respectively [41,58]. Moreover, our results agree with the earlier reports that continuous root growth under water limitation conditions is essential in wild watermelon regardless of growth conditions or development stage [23].

Watermelon has been found to cope with drought stress in several ways, including root elongation, photosystem II protection, regulation of abscisic acid (ABA), melatonin and citrulline levels, wax, osmoprotectants, and antioxidant accumulation, as well as changes in the transcriptome and proteome [4,23,24,29,35,37,59]. The drought-induced polypeptide (DRIP-1) gene, an essential enzyme in citrulline biosynthesis, plays a vital role in the drought tolerance of wild watermelon [24,34]. The root system was suggested to be involved in feeling drought by releasing a signaling molecule to the shoot and inducing the transcript of *DRIP-1* [34]. In another study, the Ran GTPase *CL*Ran1 gene, functioning in cell division and proliferation, was described as a potential enhancer of primary root growth under drought stress in wild watermelon [23,28]. Also, Nanasato et al. [32] stated that the Cytochrome b561 (CLb561A and CLb561B) and Ascorbate Oxidase (AO) are involved in drought and high light tolerance in the leaves of wild watermelon. Similarly, Akashi et al. [33] proposed Metallothionein Type-2 (CLMT2) as a significant contributor to wild watermelon survival under extreme drought and high light stresses. Herein, we mapped two QTLs related to the drought tolerance on chromosomes 1 (*qNLR_Dr. Chr01*) and 2 (*qNLR_Dr. Chr02*) (Figure 3 and Figure 4), and haplotype analysis indicated *qNLR_Dr. Chr01* is likely a major contributor to the phenotypic variation in NLR in watermelon (Figure 4). Moreover, RNA-seq results suggested that 13 differentially expressed genes in this QTL might be related to the difference in drought tolerance (Figure 6). Collectively, our results provide the first understanding of the genetic control of NLR response to drought stress in watermelon.

In several studies, combined forward and reverse genetics have been applied for gene detection and mining [60,61,62,63,64,65,66,67,68,69,70,71,72,73]. Our study has joined BSA, fine mapping, transcriptome, and polymorphism to detect candidate genes associated with drought tolerance in watermelon. The fine-mapping results suggested a region of 0.93 Mbp (*Chr01: 31601839-32348964*) within the *qNLR_Dr. Chr01* as more likely to be associated with drought tolerance than *qNLR_Dr. Chr02* in the present mapping population. The RNA-seq results show that this region includes 12 significant differentially expressed genes with nonsynonymous SNPs (Figure 6). Based on their annotation and roles published in the previous literature, six genes could be further studied to shed light on the drought tolerance mechanism according to root growth. Three transcription factors (TFs) were detected, namely *Cla97C01G019010* (ZHD2), *Cla97C01G019320* (bHLH), and *Cla97C01G019330* (bZIP53-like). *Cla97C01G019010* (ZHD2) encodes a zinc-finger homeobox TF, described recently to promote seminal and lateral root growth in rice by acting on the ethylene biosynthesis gene *ACS5* [74]. *Cla97C01G019320* is a homologous gene of *AT1G27660* (Pericycle Factor Type-A 5, PFA5), which is a basic helix–loop–helix (bHLH) DNA-binding superfamily protein and has been reported to control the initiation of lateral roots in Arabidopsis [75]. *Cla97C01G019330* (bZIP53-like) is homologous to *Arabidopsis* Basic Leucine-Zipper 11 (ATBZIP11). This bZIP family has been reported to be involved in the molecular regulation of several plant organs and tissues, such as vascular development [76], floral initiation and development [77], seed germination and maturation [78,79], embryogenesis [80], and photomorphogenesis [81]. Moreover, the bZIP family has been reported to regulate stress tolerance, including drought [82,83].

Three other candidate genes, *Cla97C01G018460* (L-ascorbate oxidase), *Cla97C01G018500* (U-box domain-containing 4-like protein), and *Cla97C01G018900* (Late embryogenesis abundant proteins, LEA), were also possibly involved in regulating root development or/and drought tolerance. The *Cla97C01G018460* (L-ascorbate oxidase) encoding ascorbate oxidase protein was described as essential in root development and stress tolerance in several species [84,85,86,87]. *Cla97C01G018500* (U-box domain-containing 4-like protein) is homologous to *AT2G22125* (Cellulose Synthase-Interactive Protein 1, CSI1), an armadillo repeat-containing protein (ARCP) that is required for root and anther development [88,89]. Moreover, PUB4, a similar gene to CS11, has been reported as a regulator of cell division and proliferation in the root meristem of *Arabidopsis thaliana* [90]. The overexpression of LEA proteins has been proven to play a role in stress tolerance, especially drought [91,92]. In another study, two LEA genes (*Cla015386* and *Cla009416*) were described as involved in watermelon osmotic stress tolerance [37], indicating *Cla97C01G018900* as a candidate gene for NLR response to drought.

## 4. Materials and Methods

### 4.1. Plant Materials, Experimental Design, and Phenotyping Procedures

A preliminary experiment was conducted at the seedling post-germination stage in pouches using one genotype exposed to 10, 15, and 20% PEG6000 (*w*/*v*) to determine the best phenotyping procedure. Distilled water was used as a basal solution for PEG treatments and a control (0% PEG6000). We started the root phenotyping at the 3–4 cm primary root length stage because, in general, at this stage, the watermelon root showed no lateral roots, allowing us to efficiently study the effect of drought stress (PEG treatment) on lateral root initiation (NLR). After exposure, we observed the root response within four days at four time points (1, 2, 3, and 4 days). We found that the 15% PEG6000 treatment for 4 days was the best condition to observe a clear difference in the lateral root system between stress and the control (Figure S1).

Accordingly, we phenotyped 38 watermelon accessions (Table S10) exposed to 0 and 15% PEG6000 to select mapping parents. Three independent replications were performed in a completely randomized design. The seed germination was performed as described in our published paper on watermelon root [22]. At 3–5 cm length of the primary root, 18 similar seedlings of each accession were divided equally into two groups (distilled water for the control and PEG 15% for the drought stress at pH = 6.00). The seedlings were transferred to new pouches (3 seedlings per pouch) and kept as described by Mahmoud et al. [22]. The phenotyping images were taken twice, on day 0 at 3–5 cm length and four days later. The manual process of the EZ-Root-VIS pipeline was used to analyze the roots [93,94]. To detect the roots, (i) the roots less than 50 pixels were rejected, (ii) the roots closer than 5 pixels were merged, and (iii) the terminal roots less than 20 pixels were pruned. We considered four root traits to make the final decision of drought tolerance, including the NLR, lateral root system (cm), primary root length (cm), and total root system (cm), which were calculated by subtraction between days 4 and 0. Interestingly, the NLR represented the main difference among the tested genotypes. Accordingly, the drought tolerance index (DTI) was developed for each studied genotype by dividing the NLR in control (CK, 0% PEG6000) by the NLR under drought stress (15% PEG6000). After that, we selected two extreme genotypes as mapping parents based on the NLR’s drought tolerance index (DTI), ZJU076 (drought-tolerant, wild watermelon) and ZJU196 (drought-sensitive, *Citrullus lanatus* (Thunb.) Matsumura and Nakai, domesticated watermelon).

To validate the root phenotypes of the selected tolerant (ZJU076) and sensitive (ZJU196) parents, we grew them in trays filled with soil (pool experiment). Briefly, seeds of each parent were sown (three rows, four seedlings/row) in a pool containing a sand/peat mixture (1:1 *v*/*v*). After germination, substrate moisture was maintained close to 50% (measured by soil moisture meter HH2; Delta Devices Co., Cambridge, UK) by watering with half-strengthening Hoagland solution every three days until the seedlings reached the two true leaf stage. At that stage, the drought was started by withholding water for 15 days (soil water content ≈ 2%, and the sensitive genotype was severely wilted). At that point, the plants were thoroughly watered to observe their recovery ability. One week after rehydration, the seedling’s phenotypes were observed. Seedlings with apparent green and viable stems were considered as tolerant. The maximum water-holding capacity of the substrate (substrate’s container capacity) was measured following the method described by Álvarez et al. [95].

### 4.2. Population Construction, Offspring Screening, and Sampling

The initial F_1_ and F_2_ populations from the ZJU076 (tolerant, ♂) × ZJU196 (sensitive, ♀) cross were obtained as described by Mahmoud et al. [22]. Accordingly, 484 F_2_ individuals were phenotyped in pouches with 15% PEG treatment for 4 d to allow BSA analysis. All F_2_ individuals were transferred to a hydroponic system to collect enough samples. After 10 days of continuous growth, samples of leaves were gathered and kept at −80 °C for further DNA extraction and pools sequencing.

### 4.3. DNA Extraction, Quality Detection, and Library Construction

The DNA was extracted using the cetyl trimethyl ammonium bromide (CTAB) procedure [96]. DNA quality and concentration were determined, as mentioned by Mahmoud et al. [22]. The DNA samples were bulked into the high pool and low pool by equally mixing 20 extreme F_2_ individuals representing tolerant and sensitive pools in addition to the two parent samples. DNA library construction and sequencing procedures were performed as described by Liao et al. [97].

### 4.4. Bulk Segregant Analysis Pipelines

The sequence alignment, mapping, SNP variant calling and annotation, SNP indexes, and G prime determination were conducted as described in Mahmoud et al. [22].

### 4.5. Haplotype Analysis for QTL Validation and Fine Mapping

We performed haplotype analysis to validate the detected QTLs in 305 F_2_ individuals and fine-mapped the target intervals with two F_2_ recombinants that covered both detected QTLs through the association between their phenotypic and genotypic variations. Briefly, the candidate regions were extracted from the VCF file after the SNPs calling pipelines. Hence, specific KASP primers were designed based on the variation in the low and high pools and parental genotypes. Twelve KASP primers (Table S11) were designed for the KASP assay and used for QTL validation and fine mapping. The KASP assay was conducted as described by Liao et al. [97]. Consequently, the genotyping data were transformed into a visual heatmap by changing the genotype values to ‘Allele 1’, ‘Allele 2’, and ‘Allele 1 & Allele 2’, representing the variants of ZJU196 (sensitive), ZJU076 (tolerant), and heterozygous, respectively. Later, ‘Allele 1’, ‘Allele 2’; and ‘Allele 1 & Allele 2’ were transformed to ‘0’;, ‘1’, and ‘2’ and labeled with green, yellow, and red, respectively, and presented in a heatmap.

### 4.6. RNA-Seq Methods

#### 4.6.1. Sample Preparation, Library Construction, and Sequencing

The whole roots of both parents were collected two days after 15% PEG6000 or control (distilled water) treatments with four biological replications (3 roots/replicate) and stored at −80 °C. Following the manufacturer’s guidelines, the total RNA was extracted using a TianGen kit (TianGen Biotech Co. Ltd., Beijing, China). RNA degradation and contamination were examined on 1% agarose gels, and the purity was evaluated using a NanoPhotometer^®^ spectrophotometer (IMPLEN, Westlake Village, CA, USA). The RNA Nano 6000 Assay Kit of the Bioanalyzer 2100 system (Santa Clara, CA, USA) was used to test the RNA integrity in accordance with the manufacturer’s instructions. The RNA libraries were prepared using total RNA for Illumina paired-end sequencing according to the Illumina protocol. Subsequently, the Illumina HiSeq 2000 platform (Illumina, San Diego, CA, USA) was used to sequence the library preparations, and 150 bp paired-end reads were created at Novogene Bioinformatics Technology Co., Ltd. (Beijing, China).

#### 4.6.2. RNA Data Analysis

Raw reads from fastq files were managed using in-house Perl scripts. Briefly, adaptor, ploy-N, and low-quality reads were eliminated from the raw reads to obtain clean reads. Simultaneously, Q20, Q30, and GC content of the clean reads were calculated [98]. UMI-tools v1.0.0 extracted the Unique Molecular Identifiers (UMIs), and the clean UMI reads with high quality were used in all the downstream analyses. The index of the watermelon reference genome V2 was built using Hisat2 v2.0.4, and paired-end clean reads were matched to the reference genome utilizing Hisat2 v2.0.4 [99]. UMI-tools v1.0.0 were used to deduplicate reads based on the mapping coordinate and the UMI attached to the reads [100]. The read numbers mapped to each gene were counted using HTSeq v0.9.1 [101] to quantify the gene expression. Consequently, the fragments per kilobase per million mapped reads (FPKM) were estimated for each gene according to the gene length and read count mapped to the same gene [102]. Differential expression analysis of 4 biological replications was conducted via the DESeq R package (1.18.0) [103]. The resulting *p-*values were adjusted via the Benjamini and Hochberg approach [104]. Corrected *p-*value < 0.05 and fold change (log_2_) of 1 were considered the threshold for significant DEGs. Gene ontology (GO) of the DEGs was applied using the GOseq R package [105], in which gene length bias was corrected. GO terms with corrected *p-*value < 0.05 were assigned as significantly enriched by DEGs. The enrichment statistics of DEGs in KEGG pathways (http://www.genome.jp/kegg/, accessed on 30 September 2021) were implemented using KOBAS v3.0 software [106]. For combined QTL and RNA-seq, only genes presenting adjusted *p*-values < 0.05 were considered for the significant DEGs in ZJU076 (tolerant) and ZJU196 (sensitive) within the detected QTLs. The CK and DR abbreviations in the RNA-Seq results indicate the control and drought stress treatments, respectively, while 76 and 196 represent the ZJU076 (tolerant) and ZJU0196 (sensitive) genotypes.

### 4.7. Detection of the Candidate Genes

We integrated the BSA, fine mapping, RNA-seq, and polymorphism results to detect the candidate genes. We only considered the DEGs with *p*-values < 0.05 in both CK_76 vs. DR_76 and CK_ 196 vs. D.R._ 196 within the detected QTLs by BSA. Specific candidate genes were proposed based on the genetic polymorphism, gene annotation, and their functions in root growth published in the previous studies.

## 5. Conclusions

Our study provides a substantial advancement in comprehending the genetic foundation of drought-associated QTLs and candidate gene detection in watermelon. We successfully mapped two QTLs (*qNLR_Dr.Chr01* and *qNLR_Dr.Chr02*) associated with NLR-dependent drought adaptation in wild watermelon. The integrated QTL mapping, fine mapping, transcriptome, and polymorphism suggested six candidate genes for drought tolerance in wild watermelon. Wild watermelon could be an excellent genetic material, rendering it valuable for pinpointing new alleles for resistance breeding. Moreover, identifying candidate genes associated with drought lays a path for subsequent studies on the underlying mechanism and provides valuable markers for drought tolerance breeding in watermelon.

## Data Availability

The datasets generated during and/or analyzed during the current study are available from the corresponding author on reasonable request.

## Supplementary Materials

The following supporting information can be downloaded at: https://www.mdpi.com/article/10.3390/ijms25010065/s1.
