# Supplementary material for: Integrated Bulk Segregant Analysis, Fine Mapping, and Transcriptome Revealed QTLs and Candidate Genes Associated with Drought Adaptation in Wild Watermelon
Source: Int J Mol Sci. 2023 Dec 20;25(1):65. doi: 10.3390/ijms25010065 (PMC10779233; doi:10.3390/ijms25010065)
Supplement: Supplementary file 1 [file ijms-25-00065-s001.zip › Supp Figures.pdf]

Supplementary Figures

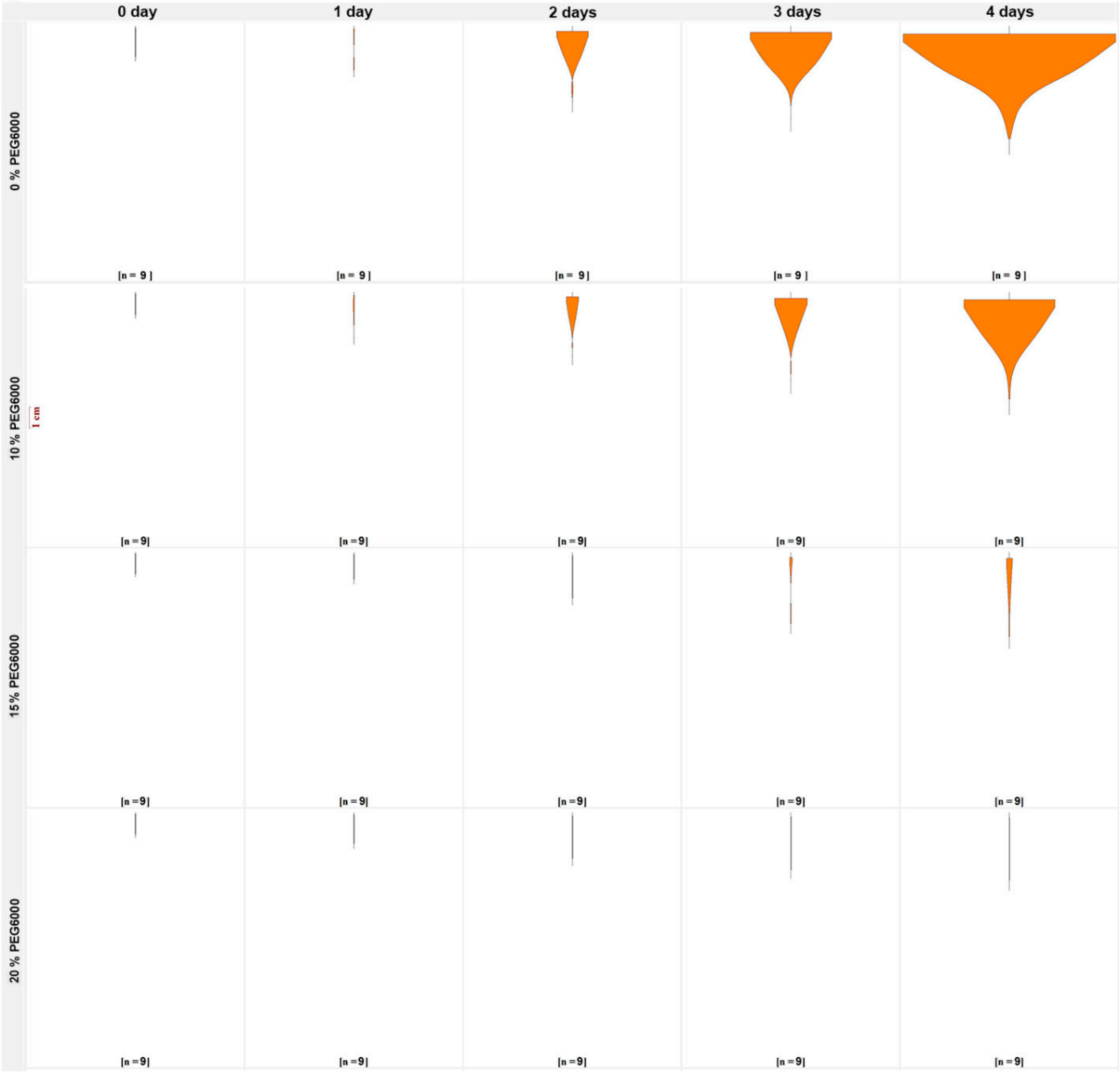

Figure S1. Lateral root profiles of one watermelon genotype as a preliminary experiment to detect the best experimental condition using 4 concentrations of PEG6000 with 4-time points.

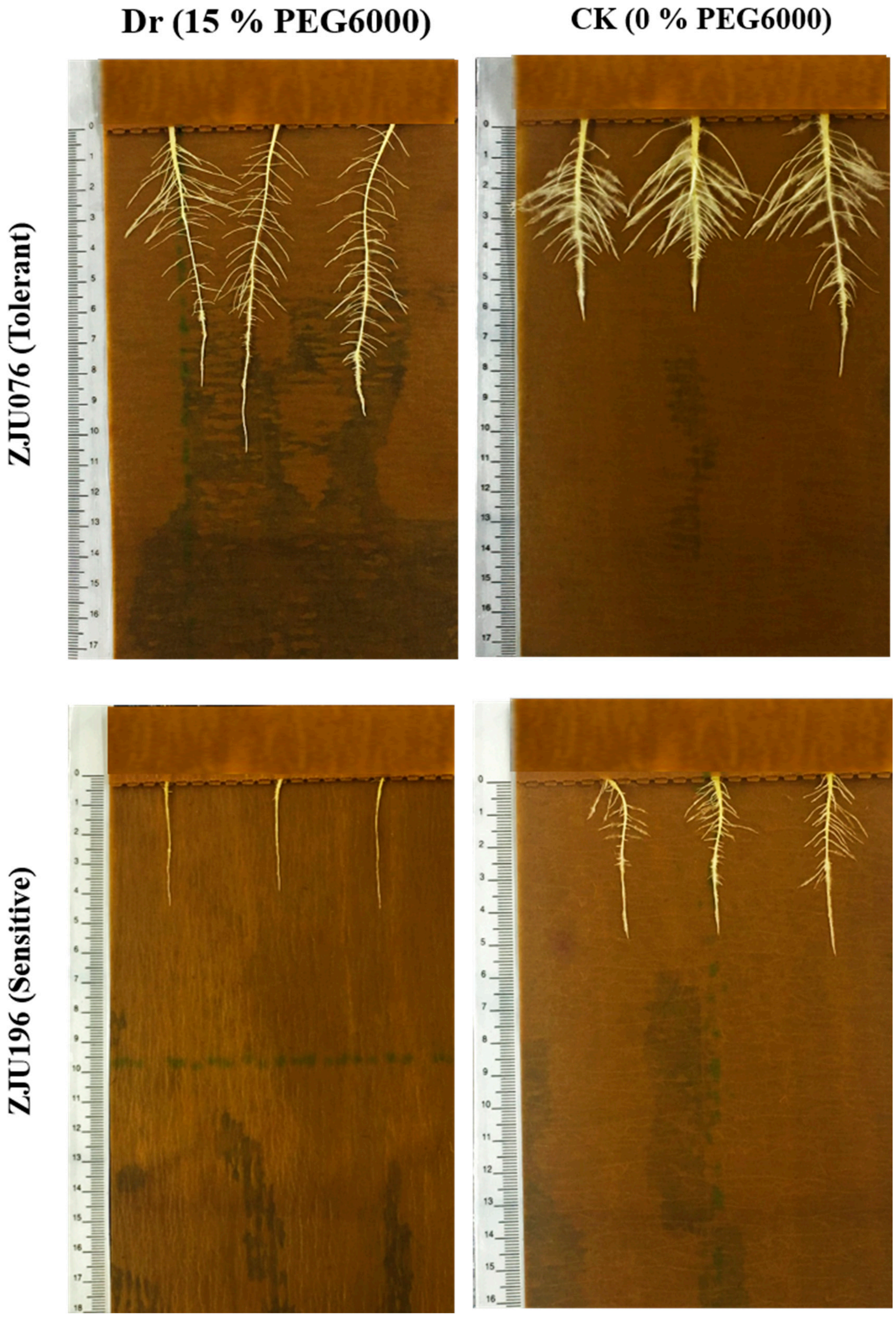

Figure S2. Root phenotypes of ZJU076 (tolerant) and ZJU196 (sensitive) after 4 days of growth under 15 % PEG6000 (Drought, Dr) or distilled water (Control, CK).

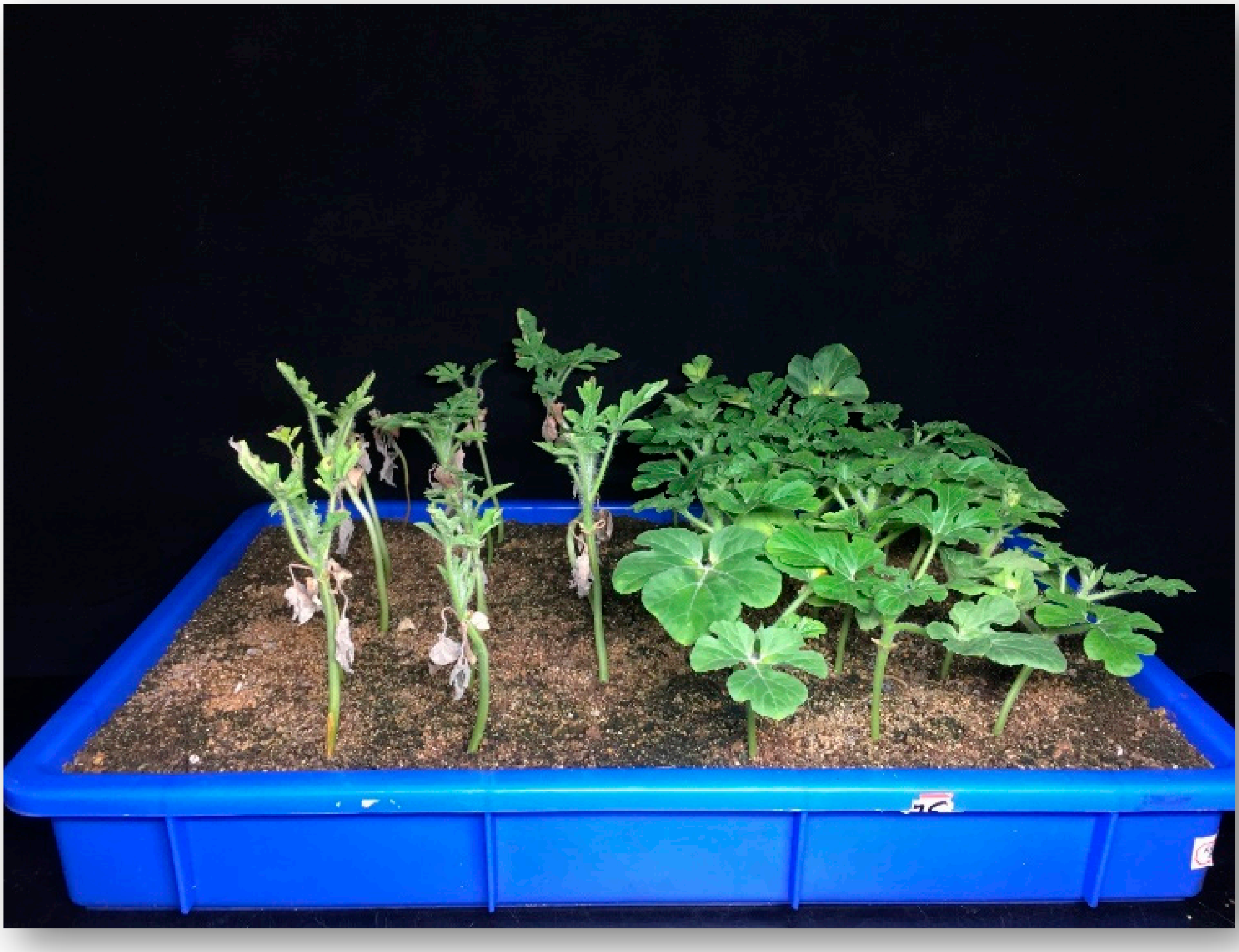

Figure S3. Phenotypes of the mapping parents one week after re-watering. Left, ZJU196; right, ZJU076.

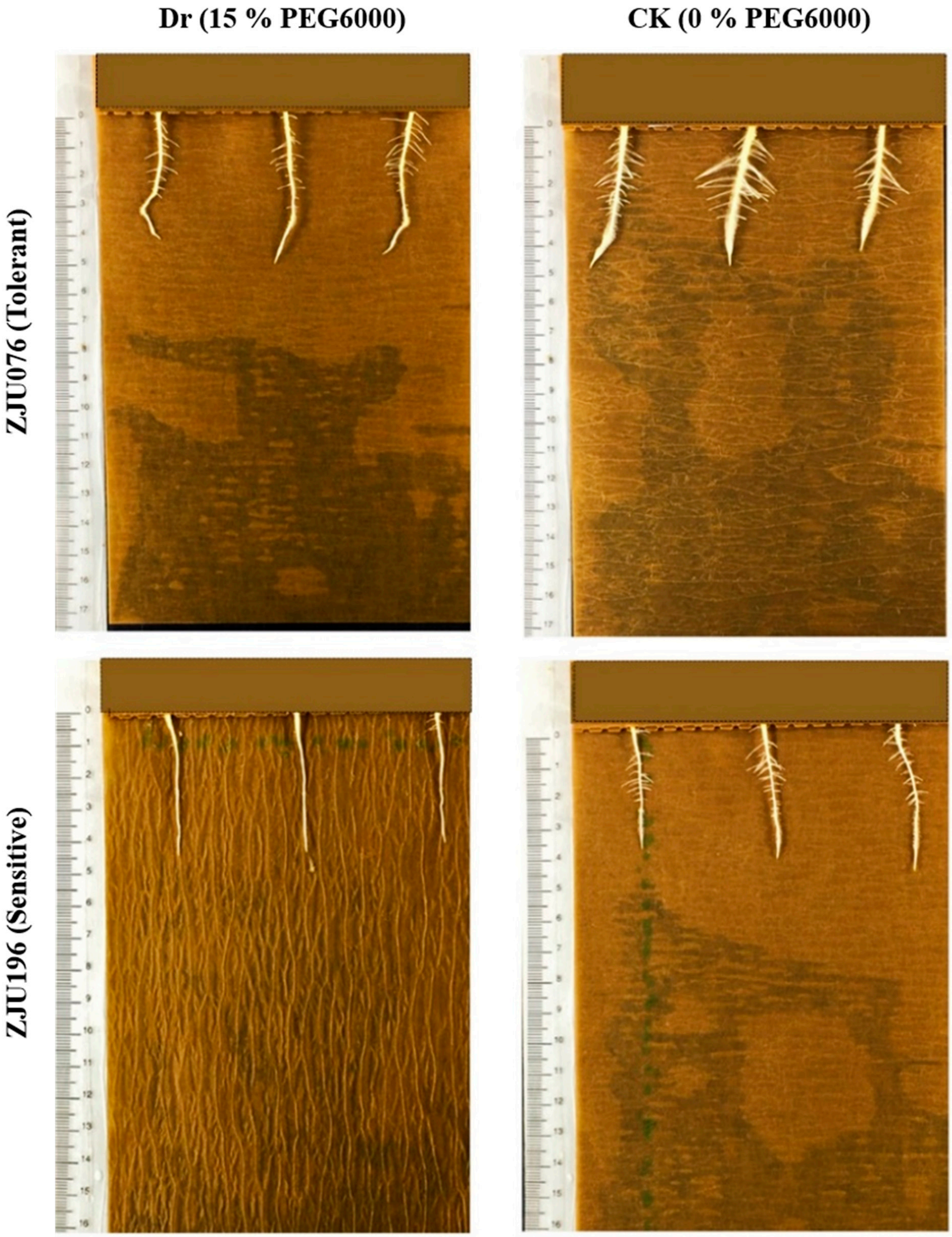

Figure S4. Root phenotypes of the selected parents in pouches after two days under under 15 % PEG6000 (Drought, Dr) or distilled water (Control, CK) treatments used for RNA-seq.

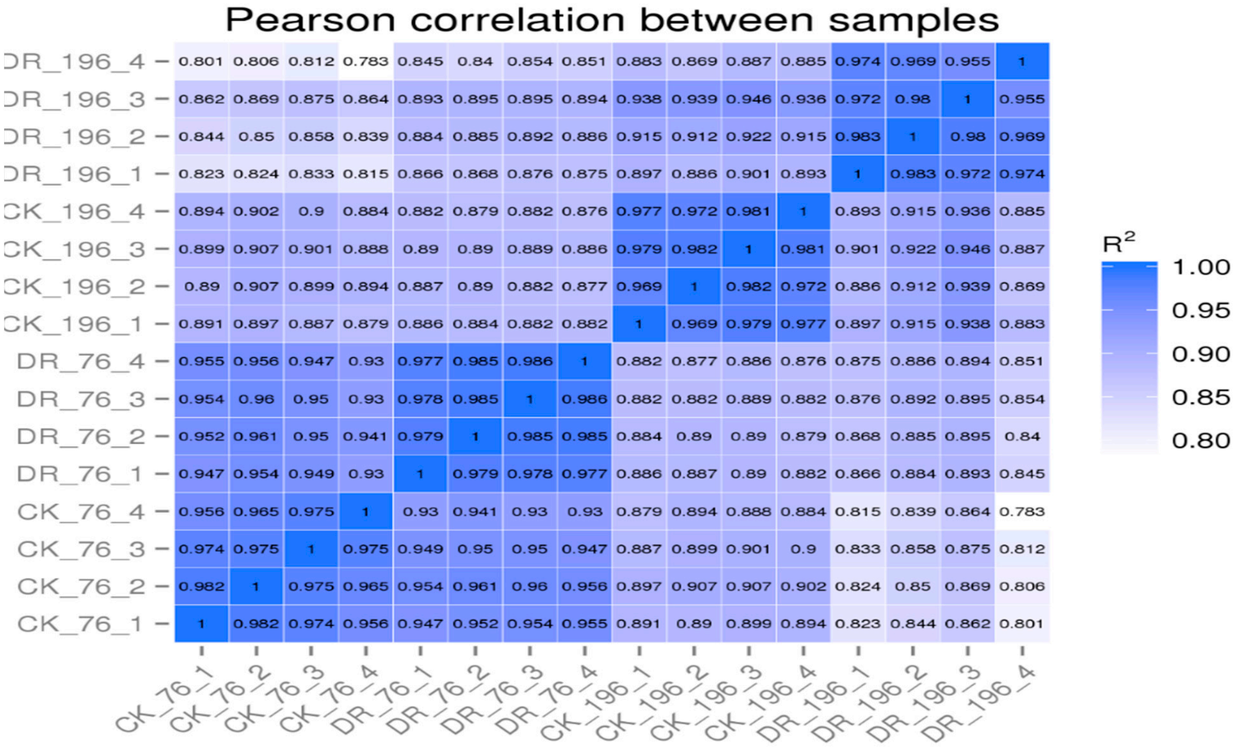

Figure S5. The Pearson correlation of gene expression levels between samples

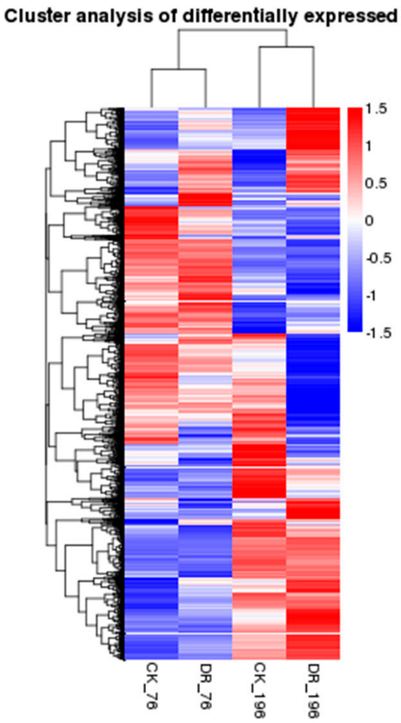

Figure S6. Hierarchical clustering analysis of the overall differentially expressed genes. The log10 (FPKM+ 1) value was normalized and transformed. CK_76, DR_76, CK_196, and DR_196 represent the control of ZJU076, drought of ZJU076, control of ZJU196 and drought of ZJU196, respectively.

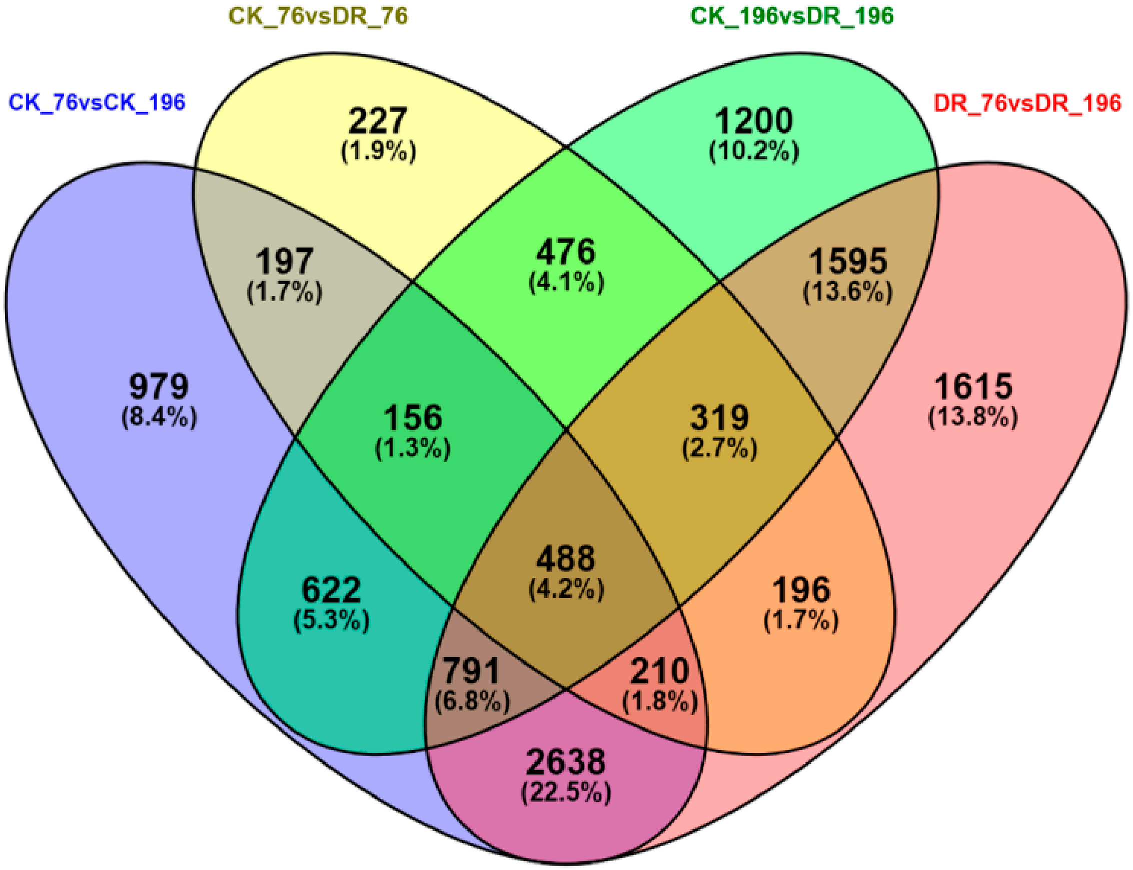

Figure S7. Venn diagrams for differentially expressed genes (DEGs) in the four comparison groups ($p$adj ≤ 0.05). The presented percentages of the 15 disjointed subgroups were calculated by dividing the DEGs in each subgroup by the total number of the unique DEGs (11709) distinguished in the four comparison groups.

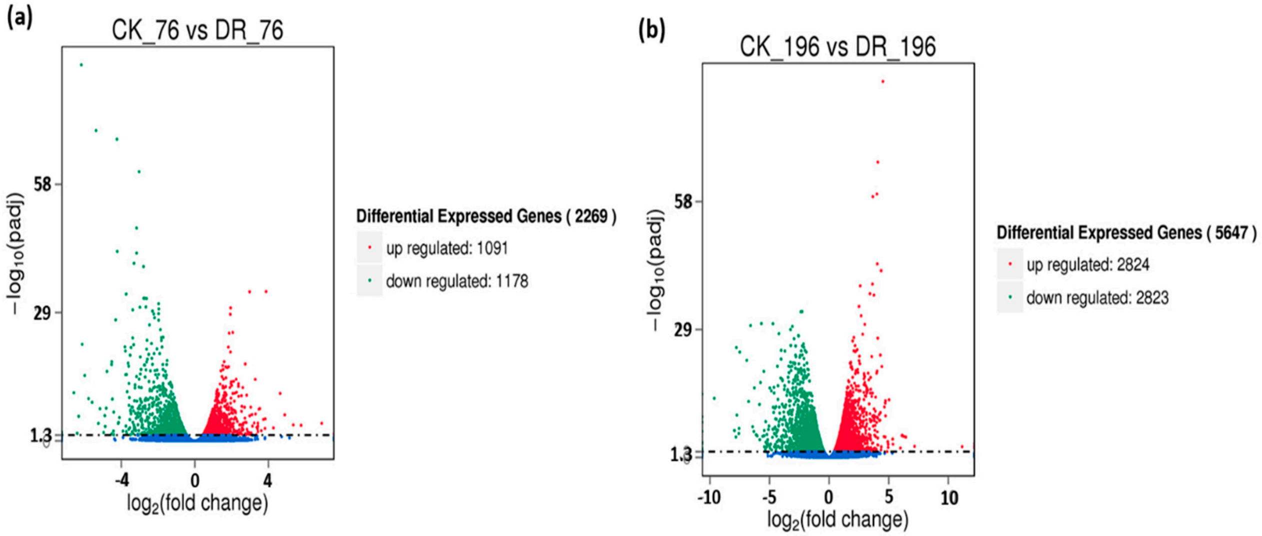

Figure S8. Volcano plots for the significant differentially expressed genes (DEGs) in response to drought. (a) CK_76 vs. Dr_76 and (b) CK_196 vs. Dr_196. X- shows the log2 (fold change) for the two treatments, while the Y-axis presents -log10 (p-adjusted). Red and green dots indicate the significant upregulated and down-regulated genes, respectively.

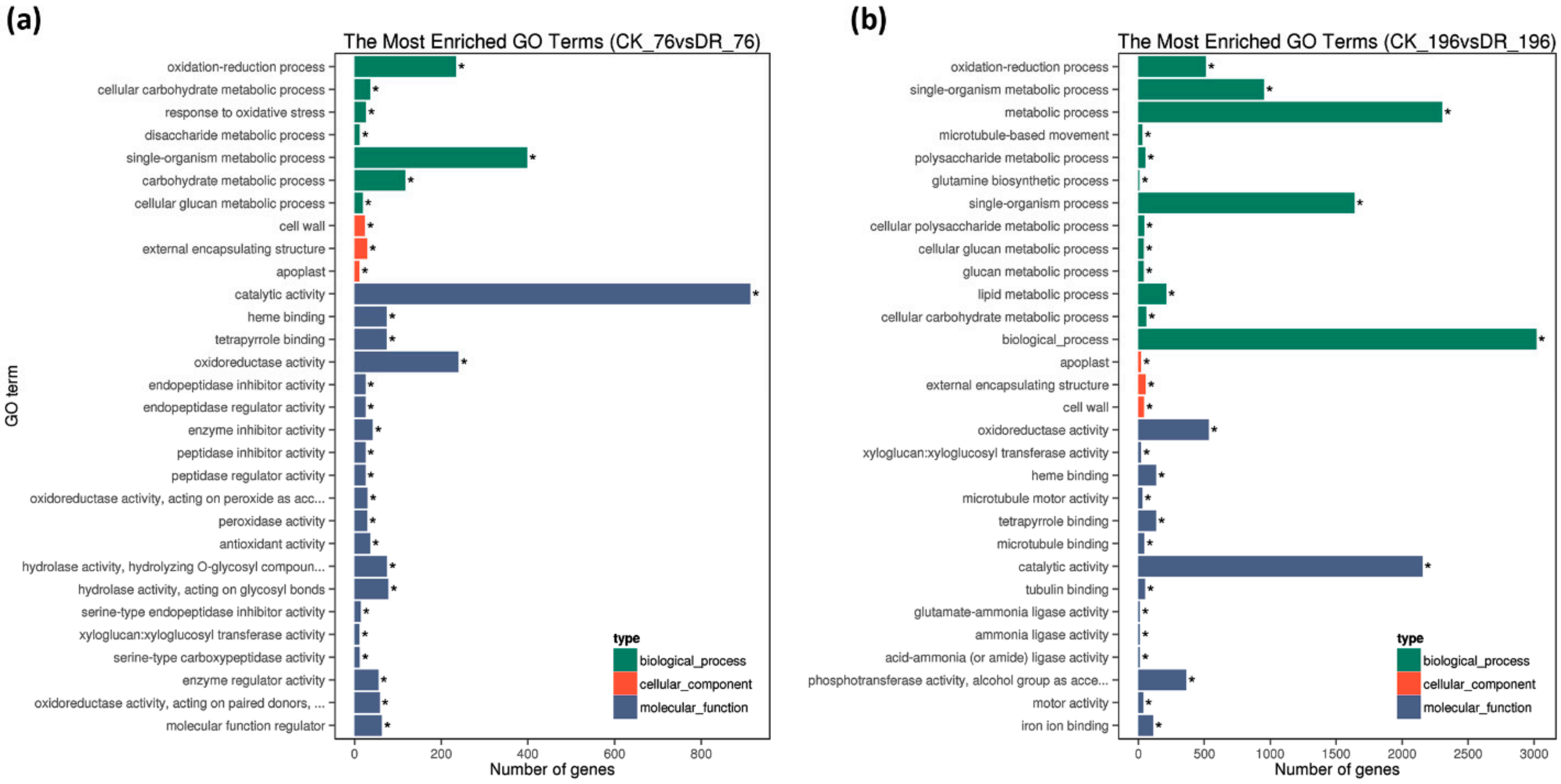

Figure S9. The most significantly enriched gene ontology (GO) terms of differentially expressed genes (DEGs) from the four comparison groups. (a) CK_76 vs. Dr_76 and (b) CK_196 vs. Dr_196. The most enriched GO terms were arranged from top to bottom based on the corrected_*p*Value ≤ 0.0

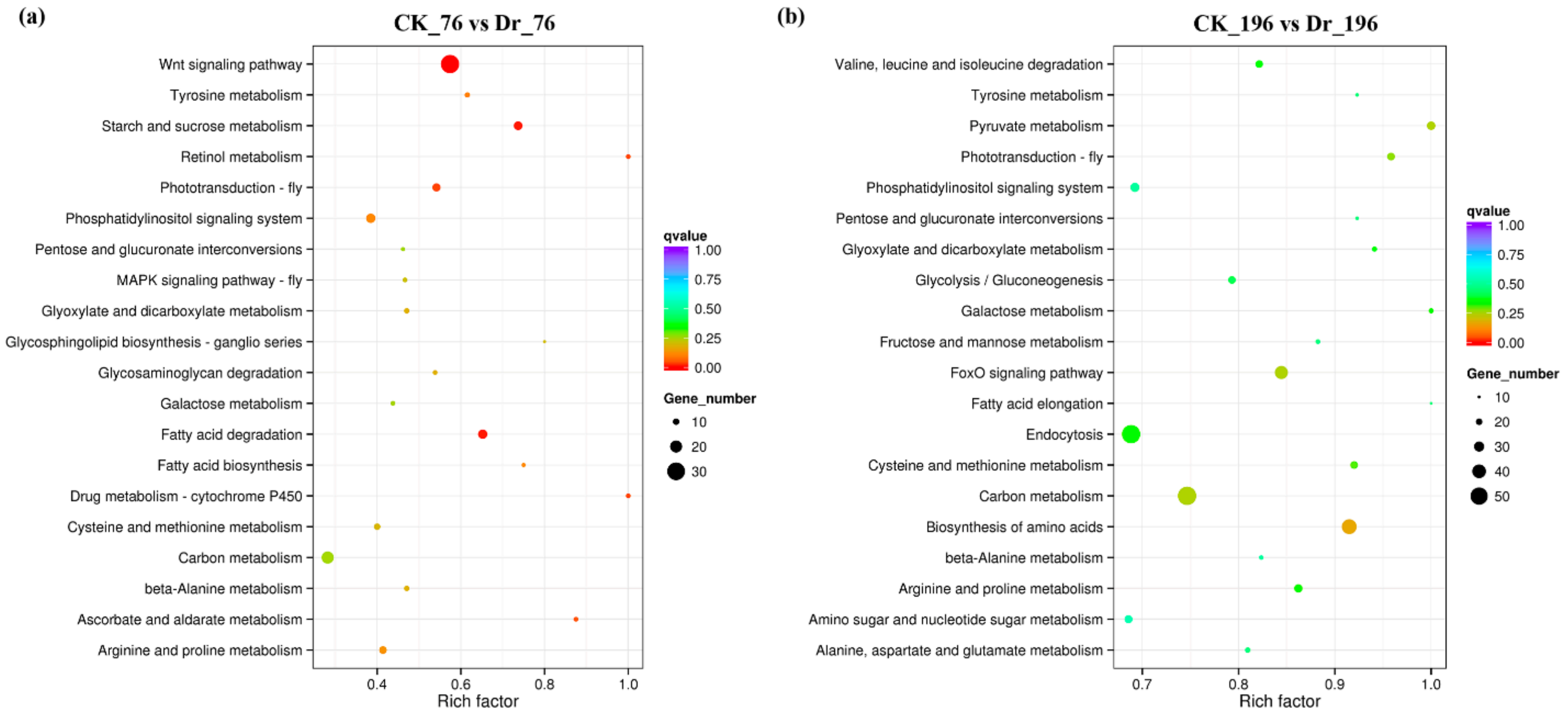

Figure S10. Statistical analysis of Kyoto Encyclopedia of Genes and Genomes (KEGG) enrichment for differentially expressed genes (DEGs). (a) CK_76 vs. Dr_76 and (b) CK_196 vs. Dr_196. The y and x-axes indicate the KEGG terms and the Rich factor, respectively. The qvalue indicates the enrichment significance in red for high and purple for low.
